# Pharmaceutical Pollutants in Urban Rats Are Linked to Zoonotic Infection Risk

**DOI:** 10.1021/acs.estlett.5c00867

**Published:** 2026-04-28

**Authors:** Anna Jonsson Sundberg, Daniel Cerveny, Federico Costa, Mike Begon, Fabio Neves Souza, Jaqueline S. Cruz, Caio Graco Zeppelini, Hernan D. Argibay, Ianei de Oliveira Carneiro, Albert I. Ko, Mitermayer G. Reis, Erin S. McCallum, Hussein Khalil

**Affiliations:** † Department of Wildlife, Fish, and Environmental Studies, 98834 Swedish University of Agricultural Sciences (SLU), SE-901 83 Umeå, Sweden; ‡ Faculty of Fisheries and Protection of Waters, South Bohemian Research Centre of Aquaculture and Biodiversity of Hydrocenoses, University of South Bohemia in Ceske Budejovice, Vodnany 389 25, Czech Republic; § Institute of Collective Health, 28111 Federal University of Bahia, Salvador 40110-040, Bahia, Brazil; ∥ Gonçalo Moniz Institute, Oswaldo Cruz Foundation, Rua Waldemar Falcão, 121, Candeal, Salvador 40296-710, Bahia, Brazil; ⊥ Institute of Integrative Biology, University of Liverpool, Biosciences Building, Liverpool L69 7ZB, U.K.; # School of Veterinary Medicine and Animal Science, Federal University of Bahia, Av. Adhemar de Barros, 500, Salvador, Bahia 40170-110, Brazil; 7 Department of Epidemiology of Microbial Diseases, 50296 School of Public Health, Yale University, New Haven, Connecticut 06520, United States; 8 Faculty of Medicine, Federal University of Bahia, Salvador 40110-040, Bahia Brazil

**Keywords:** API, ecotoxicology, zoonosis, leptospirosis, disease ecology

## Abstract

Pharmaceutical pollution is globally widespread and known to affect the biology and ecology of exposed wildlife. However, its effects on wildlife disease, including zoonotic (animal-to-human transmitted) infections, remains unclear. We examined the presence of 97 Active Pharmaceutical Ingredients (APIs) and their associations with zoonotic infections (*Leptospira* spp., Seoul orthohantavirus, *Toxoplasma gondii*, *Capillaria* spp., and *Angiostrongylus* spp.) in *Rattus norvegicus* and *R. rattus* rats, two globally distributed zoonotic reservoirs. We collected 152 rats from low-income urban communities in Salvador, Brazil, and detected APIs in 55.3% of the rats’ brain tissues, providing novel evidence of pharmaceutical uptake in a wild, terrestrial mammal. Notably, *Leptospira* infection was 91% lower in rats containing the antibiotic azithromycin, while detection of antidepressant citalopram corresponded to a 3-fold increase in *Capillaria* infection risk. Our findings show that environmental APIs absorbed by rats are associated with variation in zoonotic infection risk, representing a previously unrecognized pathway through which pharmaceutical pollution may influence disease dynamics in cities worldwide.

## Graphical abstract

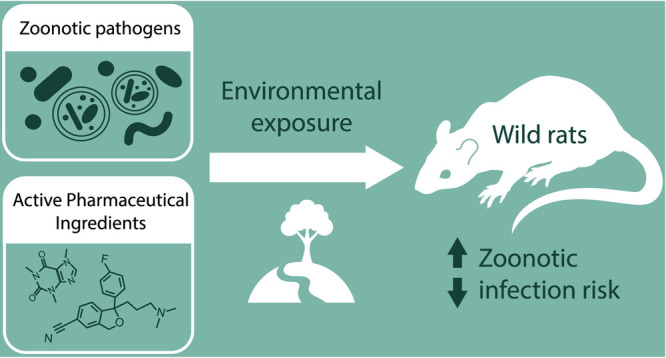

## 1 Introduction

Residues from human and veterinary pharmaceuticals have become ubiquitous in the environment, representing an emerging and increasing global challenge with implications for wildlife and human health.^1^ Emitted during their manufacturing, use, and disposal, active pharmaceutical ingredients (APIs) that produce therapeutic effects in humans or target animal populations are often incompletely metabolized by the body prior to excretion and can evade complete removal by wastewater treatment.^2^ A growing global population, rising pharmaceutical production and consumption, better access to healthcare, and improved pharmaceutical detection methods therefore all contribute to rising concerns over APIs as environmental pollutants.^3^

Our understanding of the effects of APIs on terrestrial species remains limited. Although environmental concentrations of APIs are typically lower than therapeutic doses prescribed for humans or target animals, the evolutionary conservation of many drug targets across taxa (e.g., enzymes, receptors) implies that low-level chronic exposure may subtly alter physiology, behavior, or microbiome composition in ways with meaningful ecological consequences.^4,5^ This subsequently may affect how individuals interact with their environment, including pathogen exposure and susceptibility.^6^

Changes in immunity and behavior are key pathways through which APIs can affect infection dynamics in wildlife. Antimicrobial substances, for example, can directly reduce an organisms’ infection burden and interfere with immune functions mediated by commensal microorganisms.^7,8^ The chronic presence of antibiotics in the environment provides a persistent selective pressure capable of driving antimicrobial resistance (AMR) within the host microbiome, potentially favoring resistant pathogen strains.^9^ Laboratory and field-scale studies also demonstrate that API exposure at environmentally relevant concentrations can modify wildlife behavior (e.g., neuroactive APIs),^13^ and modulate their exposure to infection via changes to host–host (e.g., aggression, grooming) and host-environment interactions (e.g., feeding habits, space-use). For example, exposure to the widespread anxiolytic pollutant oxazepam was shown to disrupt social hierarchies in brown trout (*Salmo trutta*), significantly altering aggressive interactions and stress dynamics.^14^ Such diverse and potentially contrasting effects of different APIs on wildlife physiology and behavior could have important consequences for disease ecology as well as wildlife and human health.

Approximately 60% of known human infectious disease pathogens have zoonotic origins,^15^ disproportionately affecting low-income populations that are often trapped in cycles of poverty and ill health.^16^ In these areas, an increasing access to pharmaceutical substances coupled with high human densities and inadequate sanitation have also raised concerns about environmental API pollution.^1^ Given that most global population growth by 2050 is predicted to occur in low-income urban regions,^17^ it is therefore crucial to understand how APIs in the environment can influence disease dynamics in urban zoonotic reservoirs.

Urban rats (*Rattus norvegicus* and *R. rattus*) are ideal model organisms for investigating the intersection between APIs and zoonotic transmission. They are globally distributed, omnivorous, and closely linked to aquatic environments and associated human waste and sewerage.^18^ Additionally, they are considered hyper-reservoirs: species that harbor numerous zoonotic pathogens responsible for disease and mortality in humans.^19^ Here, we evaluated the presence of 97 common APIs in rats from urban low-income communities and explored their associations with potential environmental sources of contamination and bacterial infections by *Leptospira* spp., viral Seoul orthohantavirus (SEOV) infection, and parasitic infections by *Toxoplasma gondii*, *Capillaria* spp., and *Angiostrongylus* spp. Our study provides evidence for pharmaceutical contamination as a previously unrecognized environmental driver of zoonotic infection patterns in urban wildlife, with potential implications for disease risk in vulnerable communities worldwide.

## 2 Materials and Methods

### 2.1 Experimental Design and Study Population

We collected *Rattus norvegicus* and *R. rattus* rats from seven slum communities in the city of Salvador-Bahía (692,819 km^2^) in northeastern Brazil (Figure S1): Pau da Lima (PdL), Alto do Cabrito (AdC), Marechal Rondon (MR), Sete de Abril (SdA), Nova Sussuarana (NS), Rio Sena (RS), and Nova Constituinte (NC). The sampling was conducted through three longitudinal and cross-sectional rodent sampling projects between 2013 and 2023 (sampling campaigns (A) 2016, (B) 2021–2023, and (C) 2013–2018) using food-baited live-traps. Captured rats were anaesthetised, euthanised and transported to a laboratory for external examination and dissection. More information on study areas, rat sampling protocols and sample storage is included in the Supporting Information.

All rat trapping and handling followed protocols validated and approved by the Ethical Committee of the Animal Use (CEUA) protocols 003/2012, 019/2016, and Committee of Ethics in Research of the Institute of Collective Health–Federal University of Bahia (UFBA), no. 041/17, no. protocol 2.245.914.

### 2.2 API Analysis

Brain tissue sample preparation followed the protocol described by McCallum et al.^20^ We analyzed samples for 97 APIs by liquid chromatography-tandem mass spectrometry (LS-MS/MS). The panel, previously validated at environmentally relevant tissue concentrations, represents diverse drug classes (antibiotics, antidepressants, antipsychotics, and others) that are widely detected in wastewater globally^1^ (Table S1). Quality assurance and control for the performance, repeatability, and recovery of the analytical method were documented during method development.^21,22^ We determined limits of qualification (LOQs) for each target analyte and tissue sample.^21^ We favored brain tissues over other tissue samples as the subject of pharmaceutical screenings to optimize the detection of behavior-modulating compounds that act via the central nervous system. In the control (blank) samples, no target compounds were detected above the LOQ, and measured concentrations in test samples were blank-corrected. Additional details can be found in the Supporting Information (Supporting Information, Table S1).

### 2.3 Pathogen Screenings

#### 2.3.1 Quantitative Real-Time Polymerase Chain Reaction (qPCR) and Kidney Imprints for Detection of *Leptospira* spp

We detected *Leptospira* sp. using a combination of qPCR targeting the lipL32 gene and kidney immunofluorescence imprints;^23,24^ infection status was assigned primarily based on qPCR results, with kidney imprints used when qPCR data were unavailable, as they are concordant with but less sensitive than qPCR.^23,25^

#### 2.3.2 Enzyme-Linked Immunosorbent Assay (ELISA) for Detection of Seoul Orthohantavirus (SEOV)

We determined SEOV positivity using a modified enzyme-linked immunosorbent assay (ELISA) based on a hantavirus recombinant nucleoprotein antigen.^26^

#### 2.3.3 Polymerase Chain Reaction (PCR) for Detection of *Toxoplasma gondii*

We tested rat urine for *Toxoplasma gondii* DNA through nested-PCR targeting the ITS1 region of *T. gondii*, with sensitivity and specificity validated against serial dilutions and closely related taxa both in silico and empirically.^27,28^

#### 2.3.4 Helminth Egg and Larvae Detection

We determined infections by *Angiostrongylus* and *Capillaria* helminths using a modified Hoffman sedimentation technique applied to formalin-fixed feces, confirmed by detection of adult parasites in tissue samples.^31,32^

### 2.4 Statistical Analysis

We conducted all statistical analyses in R v.4.2.3 R Core,^34^ using a significance threshold of *p* < 0.05. We tested for co-occurrence among the six most frequently detected APIs using pairwise chi-square tests. We evaluated associations between infection status (*Leptospira* spp., SEOV, *T. gondii, Capillaria* spp., *Angiostrongylus* spp., and *Capillaria* and *Angiostrongylus* spp. combined) and API detection using binomial GLMs, with two models fitted per pathogen: one testing for the detection of any API and another for the detection of each of the six most frequently detected APIs. All final models were determined by (a) selection of explanatory variables indicated by previous literature,^35–37^ followed by (b) selection of pharmaceutical focal predictors, with final models chosen based on AICc and tested for multicollinearity (VIF < 4). We used a similar GLM-based approach to identify environmental predictors of API detection, using a panel of 25 environmental quality variables. To account for multiple comparisons, we applied Benjamini–Hochberg (BH) false discovery rate correction across pharmaceutical predictors within each pathogen-API model^40^ (see Supporting Information).

## 3 Results

### 3.1 APIs Detected in Rat Brain Tissues

To test whether urban rats uptake APIs from their environment, we screened the brain tissues of 152 rats (4 *R. rattus*, 127 *R. norvegicus*, 21 unknown spp.) from seven low-income communities in Salvador, Brazil (Figure 1), collected in 2009–2020 for 97 APIs with a broad functional range (Table S1). We selected our study areas based on their high rate of rat infestation^41^ and zoonotic infection prevalence,^31,42,43^ combined with a high risk of pharmaceutical contamination due to inadequate sanitation. Residents in these communities generally have a low socioeconomic status and live in poor-quality houses with insecure tenure,^44^ with untreated wastewater and sewerage flowing in open canals that regularly flood during heavy rainfall.

**Figure 1:**
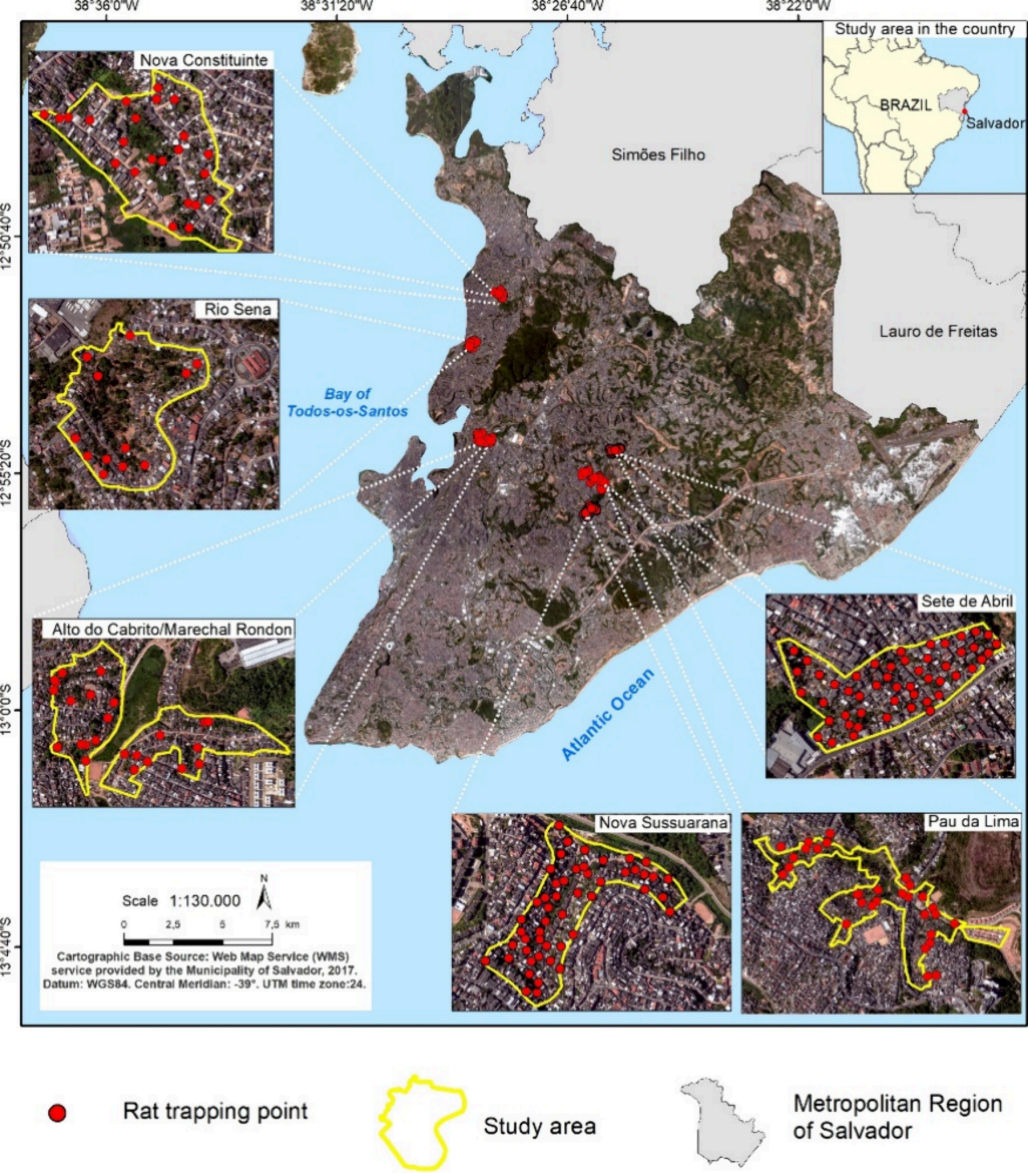
**Rats collected in Salvador, Brazil.** Trapping location GPS coordinates were unavailable for four rats, which have been omitted from the map.

The outcomes of our pharmaceutical screening are listed in Table 1. We detected a total of 18 different APIs in 84 (55.26%) of the rats, confirming that a majority of these rats had absorbed pharmaceuticals from their environment. Of rats with detectable APIs, 29.76% contained mixtures of multiple compounds (Table S2). The detected compounds spanned multiple drug classes, including antibiotics, antidepressants, antipsychotics, stimulants, and antihistamines (Table 1). The highest detection frequency was observed for citalopram, a selective serotonin reuptake inhibitor (SSRI) antidepressant, with levels greater than the LOQ in 26% of captured rats (Table 1).

**Table 1:** Pharmaceuticals Detected in the Brain Tissues of Wild Rats^a^

			Detected concentration range (ng g^–1^)		
Compound	Intended function	Detection count (% of rats)	Mean	Median	Max
Amiodarone	Antiarrhythmic	1 (1%)	0.54	0.54	0.54
Azithromycin	Antibiotic	14 (9%)	0.21	0.68	3.19
Benzoylecgonine	Stimulant	1 (1%)	0.51	0.51	0.51
Caffeine	Stimulant	9 (6%)	3.46	57.62	390.06
Carbamazepine	Anticonvulsant	6 (4%)	0.13	1.15	5.90
Citalopram	Antidepressant	40 (26%)	0.07	0.08	0.19
Clindamycin	Antibiotic	9 (6%)	0.06	0.07	0.11
Desloratadine	Antihistamine	1 (1%)	5.75	5.75	5.75
Diclofenac	Anti-inflammatory	1 (1%)	0.52	0.52	0.52
Donepezil	Dementia treatment	22 (14%)	0.07	0.18	0.63
Fexofenadine	Antihistamine	2 (1%)	0.21	0.21	0.35
Glibenclamide	Diabetes treatment	2 (1%)	0.29	0.29	0.31
Haloperidol	Antipsychotic	8 (5%)	0.10	0.25	0.74
Levomepromazine	Antipsychotic/analgesic	1 (1%)	0.11	0.11	0.11
Tamoxifen	Antiestrogen	1 (1%)	0.06	0.06	0.06
Telmisartan	Antihypertensive	1 (1%)	0.09	0.09	0.09
Tramadol	Antidepressant/analgesic	1 (1%)	0.07	0.07	0.07
Trimethoprim	Antibiotic	3 (2%)	1.12	0.87	1.30

a Presence and concentration of 18 Active Pharmaceutical Ingredients (APIs) detected in the brain tissues of wild-caught rats captured in Salvador, Brazil (*n* = 152). Tissue concentrations detected in rat brain tissue (0.07–54 ng g^–1^ wet weight) are substantially below typical clinical therapeutic plasma concentrations, consistent with sub-therapeutic environmental exposure.

### 3.2 API Detection Is Associated with Variation in Infection Risk

We tested whether the detection of each of the six most frequently detected compounds, as well as the detection of any API, was associated with infection by five locally prevalent pathogens. Our panel of target pathogens includes only taxa with zoonotic species and represents diverse pathogen types and transmission routes, including *Leptospira* spp. bacteria, Seoul orthohantavirus (SEOV), the protozoan parasite *Toxoplasma gondii*, and nematodes in the *Capillaria* and *Angiostrongylus* genera (see Supporting Information for details on pathogens).

We observed distinct patterns of association between API detection and infection status that varied by pathogen (Figure 2). The probability of *Leptospira* infection was 74% lower in rats with any API > LOQ, regardless of pharmaceutical identity (OR = 0.26, 95% CI [0.10, 0.70], *p* = 0.007). *Leptospira* infection was also 91% lower in rats with detectable azithromycin (OR = 0.09, 95% CI [0.01, 0.85], *p* = 0.035, *q* = 0.140), and this association remained when azithromycin detection was excluded from the any-API variable (OR = 0.37, 95% CI [0.14, 0.95], *p* = 0.038, Table S3), together suggesting a broader pharmaceutical effect on *Leptospira* infection risk. Rats containing quantifiable citalopram were more than three times as likely to be infected by *Capillaria* nematodes (OR = 3.41, 95% CI [1.12, 10.42], p = 0.031, q = 0.063) and more than twice as likely to be infected by SEOV as other rats, though the latter result was near-significant (*p* = 0.065). The azithromycin–*Leptospira* and citalopram–*Capillaria* associations represent marginal trends after BH correction and should be interpreted as hypothesis-generating findings. A near-significant association was also observed between clindamycin detection and *Angiostrongylus* infection risk (OR = 8.67, 95% CI [0.87, 86.1], *p* = 0.065), though this was based on a small sample (*n* = 33). No pharmaceutical predictors were retained in the final models for the combined *Capillaria/Angiostrongylus* infection. (Figure 2).

**Figure 2:**
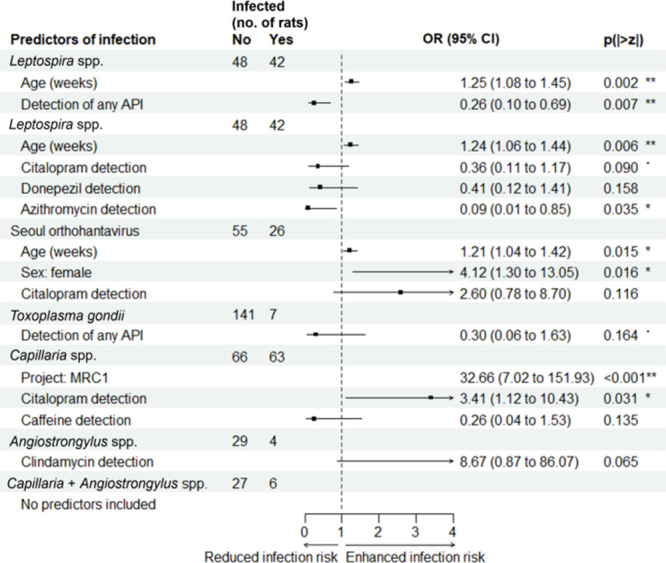
**Associations between API detection in rats and their infection risk reveal potential pharmaceutical effects.** Final binomial regression (GLM) models testing for the effect of API detection at two levels of resolution (probability of detecting any API and probability of detecting each of the six most frequently detected APIs) on infection risk by five infectious agents (point = odds ratio (OR) estimate, line = 95% confidence interval (CI), · = *p* < 0.10, * = *p* < 0.05, ** = *p* < 0.01). Final models including no pharmaceutical predictors have been excluded.

The *Leptospira* infection risk increased with rat age, and *Capillaria* infections were more likely in female than in male rats. Rats captured in 2022–2023 through sampling campaign B were more likely to have *T. gondii* infections than other rats (Figure 2).

### 3.3 Associations with Potential Environmental Sources

We explored whether environmental features of the rats’ surroundings could predict their probability of containing detectable APIs using an approach similar to that described above. We recorded the presence of environmental features previously shown to be associated with rat infestation and zoonotic risk (e.g., open sewers, trash, food waste)^45^ within a 20-m radius of all trapping points and used this data to model pharmaceutical detection.

Each of the six most frequently detected APIs had unique environmental predictors, with no consistent predictors across all analytes (Figure 3), suggesting that environmental sources and routes of pharmaceutical absorption in rats may vary by compound.

**Figure 3:**
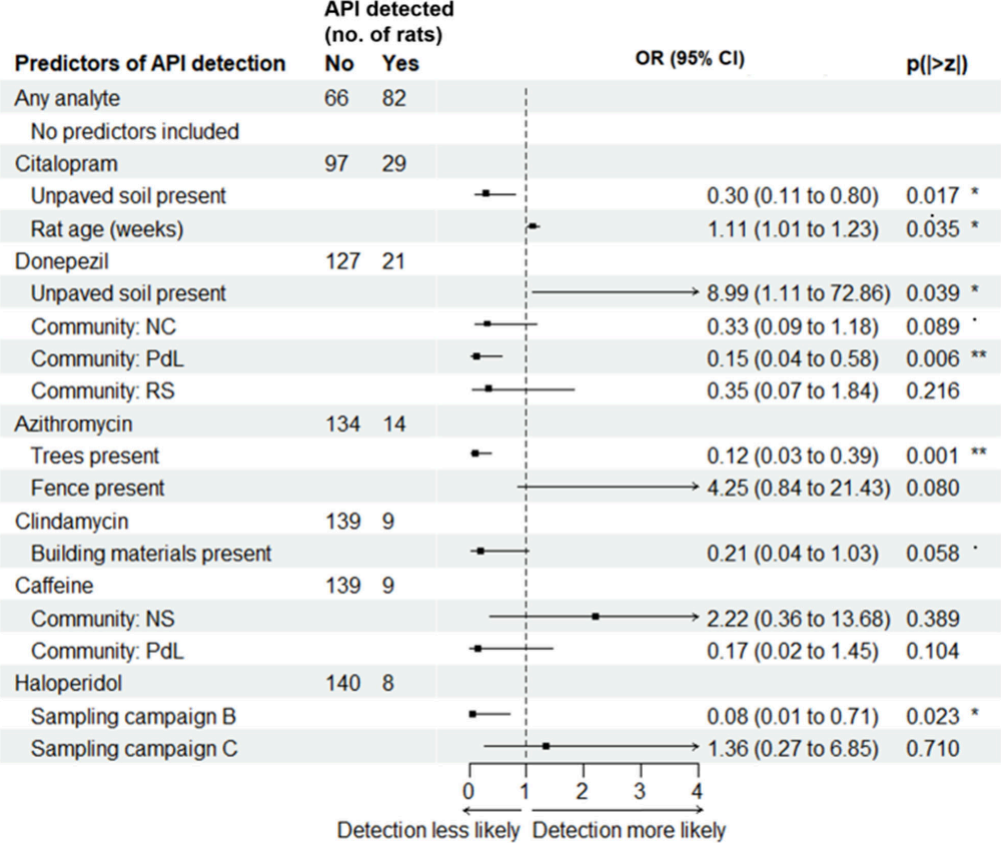
**Environmental predictors of pharmaceutical detection in rats.** Final binomial regression (GLM) models explaining the detection of APIs within rat brain tissues (point = odds ratio (OR) estimate, line = 95% confidence interval (CI), * = *p* < 0.10, ** = *p* < 0.05, *** = *p* < 0.01). All environmental predictors were measured within a 20 m radius of rat trapping points.

## 4 Discussion

We provide a novel investigation into the presence of APIs in wild, terrestrial mammals and their association with zoonotic infection patterns using urban rats from Salvador, Brazil. We found that the majority of the 152 rats (55.26%) in our study contained APIs in their brain tissues, with more than one-third of these positive samples containing mixtures of multiple pharmaceuticals. In addition, we discover links between API detection in rats and their infection risk, with rats containing the antibiotic azithromycin being 91% less likely to carry *Leptospira* bacteria, a major zoonotic pathogen that is responsible for approximately one million severe human leptospirosis cases annually.^46^ Taken together, our study indicates that pharmaceutical contamination may alter infection patterns in important zoonotic reservoirs (Figure 4).

**Figure 4:**
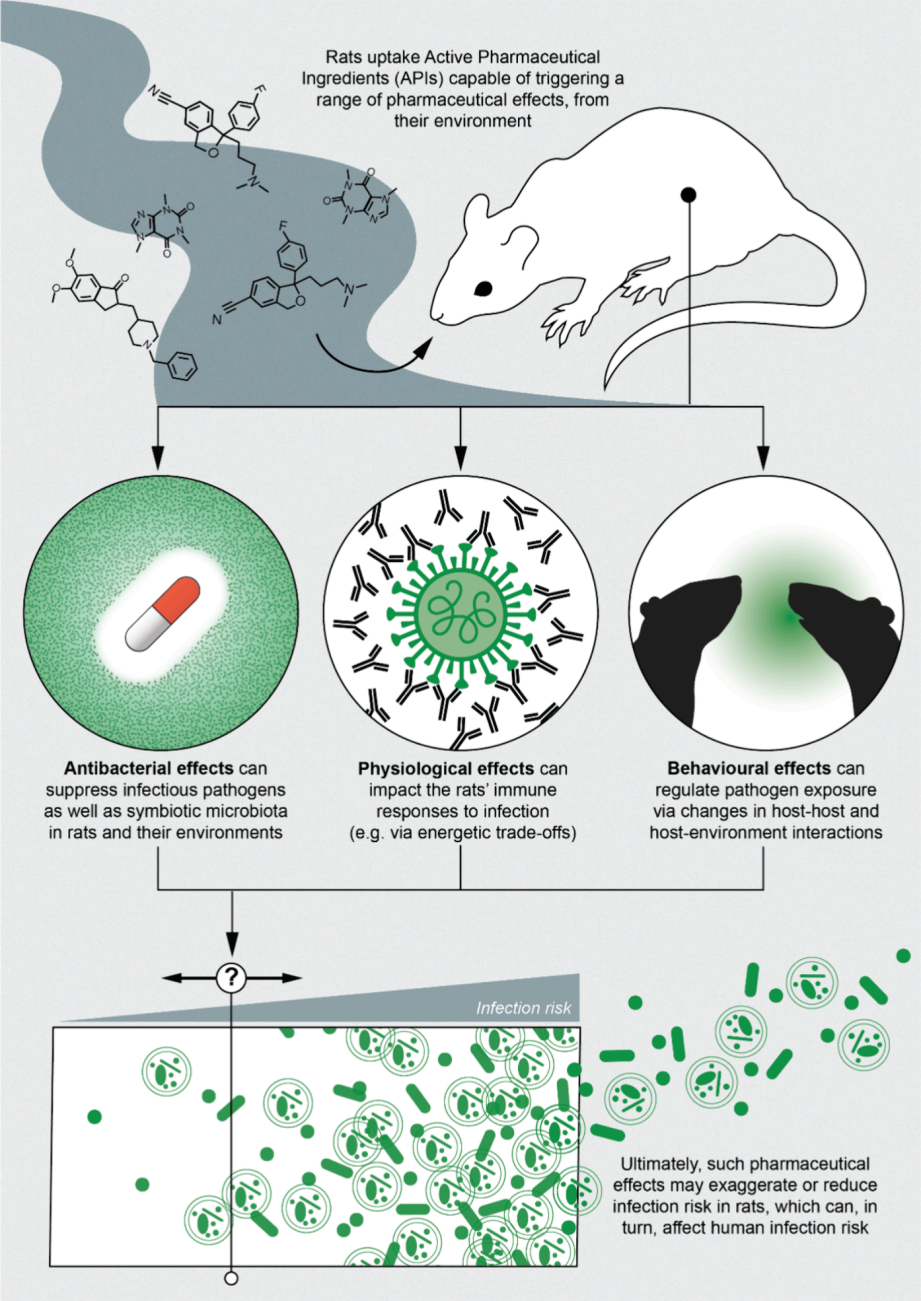
**Potential mechanisms of infection regulation in wild rats exposed to pharmaceuticals.** Urban rats, which are closely associated with human activities including waste and sewerage, can uptake various Active Pharmaceutical Ingredients (APIs) from their environment. Once transported into the rats’ tissues, we hypothesize that these compounds trigger a range pharmaceutical effects with potential consequences for the rats’ infection risk. For zoonotic pathogens, this can also influence human disease risks.

The relationship between pharmaceutical contamination and zoonotic infection risk represents a potentially important pathway through which environmental pollution may affect public health in urban environments, globally. The reduced *Leptospira* infection risk in rats with detectable azithromycin concentrations is consistent with a protective antibiotic effect. The mechanisms could involve direct antibacterial activity against *Leptospira*,^47,48^ immunomodulatory effects via gut microbiota alterations,^49,50^ and reduced environmental *Leptospira* viability, given the high persistence of azithromycin in soil^51^ (a key transmission reservoir in urban slums).^52^ Notably, infection risk was also lower in rats with any detectable API even after excluding azithromycin, suggesting broader pharmaceutical effects beyond this specific antibiotic.

In contrast, rats with detectable levels of the antidepressant citalopram showed a 3-fold increase in *Capillaria* parasite infections. This increase in infection risk by a neuroactive pharmaceutical suggests that API pollution may have cascading effects on disease ecology. As a selective serotonin reuptake inhibitor, citalopram could modify rat behavior through increased boldness and locomotor activity^53,54^ (although some studies report context-dependent or opposite effects in invertebrates at environmentally relevant concentrations^55^), potentially expanding their environmental exploration and increasing encounters with contaminated food and water sources.

Our results provide initial evidence for a pathway linking pharmaceutical pollution with disease risk in wildlife, highlighting the need for additional studies to unravel the mechanisms through which APIs modulate zoonotic infections in urban rats. Specifically, laboratory challenge experiments at environmentally relevant API concentrations could test whether azithromycin directly reduces *Leptospira* colonization in rats and whether citalopram-induced behavioral changes (e.g., increased foraging range) increase *Capillaria* encounter rates. Fine-scale tracking of individual rat movements would allow for direct testing of whether spatial exposure heterogeneity drives the associations observed. Multicity investigations in comparable low-income urban settings across the Global South will be necessary to determine whether these patterns are generalizable to the urban zoonotic landscapes.

There are several limitations to our study. First, our observational cross-sectional design cannot confirm causality or the specific mechanisms by which APIs influence infection risk. Second, our estimates of pharmaceutical uptake are conservative, as we analyzed only brain tissue for a subset of 97 out of approximately 3,000 APIs currently in use, and precollection metabolism likely causes underdetection.^56^ Third, while unmeasured variables could act as confounders, our study design and local ecology mitigate spatial bias. Finally, although our sampling spanned a decade, we controlled for rat age, sex, and sampling campaign to account for demographic and seasonal differences.

As commensal rodents serve as key reservoirs for numerous zoonotic pathogens in cities globally, the potential of environmental pharmaceuticals to alter infection patterns in these animals may have important implications for the zoonotic risk. This is especially relevant for vulnerable urban communities in rapidly developing regions with inadequate sanitation, pharmaceutical access, and high human–wildlife contact. Our study highlights the need for interdisciplinary research to understand the behavioral and physiological mechanisms underlying API-pathogen associations, examine potential long-term consequences such as antimicrobial resistance, and develop targeted interventions to mitigate risks while potentially leveraging beneficial effects.

## Supplementary Material







## Data Availability

All data, code, and materials used in the analyses are available in the .

## Notes

Target pathogen overviews, experimental design details, API analytical methods, pathogen detection protocols (qPCR/kidney imprints for *Leptospira* spp., ELISA for SEOV, nested-PCR for *Toxoplasma gondii*, and helminth sedimentation), statistical analysis procedures, API limits of quantification, co-occurrence analyses, GLM model selection results, and variance inflation factors (PDF)
Raw data for pharmaceutical screenings (CSV)
Raw data for rat physiological, demographic, and environmental assessments (CSV)
