# Supplementary material for: Pharmaceutical Pollutants in Urban Rats Are Linked to Zoonotic Infection Risk
Source: Environ Sci Technol Lett. 2026 Apr 28;13(5):656–63. doi: 10.1021/acs.estlett.5c00867 (PMC13173639; doi:10.1021/acs.estlett.5c00867)
Supplement: Supplementary file 1 [file ez5c00867_si_001.pdf]

Supplementary Materials for

Pharmaceutical pollutants in a wild, urban rat population and link to zoonotic infection risk

Anna Jonsson Sundberg[1*], Daniel Cerveny[1,2], Federico Costa[3,4], Mike Begon[5], Fabio Neves Souza[3], Jaqueline S. Cruz[4], Caio Graco Zeppelini[1,3], Hernan D. Argibay[3,4], Ianei de Oliveira Carneiro[3,6], Albert I. Ko[4,7], Mitermayer G. Reis[4,7,8], Erin S. McCallum[1]† and Hussein Khalil[1,3]*†

Affiliations

[1] Department of Wildlife, Fish, and Environmental Studies, Swedish University of Agricultural Sciences (SLU), SE-901 83, Umeå, Sweden.

[2] Faculty of Fisheries and Protection of Waters, South Bohemian Research Centre of Aquaculture and Biodiversity of Hydrocenoses, University of South Bohemia in Ceske Budejovice, Vodnany 389 25, Czech Republic.

[3] Institute of Collective Health, Federal University of Bahia, Salvador 40110-040, Bahia, Brazil.

[4] Gonçalo Moniz Institute, Oswaldo Cruz Foundation, Rua Waldemar Falcão, 121, Candeal, Salvador 40296-710, Bahia, Brazil.

[5] Institute of Integrative Biology, University of Liverpool, Biosciences Building, Liverpool, L69 7ZB, UK.

[6] School of Veterinary Medicine and Animal Science, Federal University of Bahia, Av. Adhemar de Barros, 500, Salvador, Bahia 40170-110, Brazil.

[7] Department of Epidemiology of Microbial Diseases, School of Public Health, Yale University, New Haven, CT, United States.

[8] Faculty of Medicine, Federal University of Bahia, Salvador, Bahia, Brazil.

* Corresponding author
† Shared last authorship

This PDF file includes:

Supplementary Text
Tables S1 to S32

Other Supplementary Materials for this manuscript include the following:

Data S1 to S2

Supplementary Text

Target pathogen overview

We selected our panel of target pathogens to include only taxa with zoonotic species documented in urban Salvador, representing diverse pathogen types (bacterial, viral, protozoan, and helminthic) and transmission routes. All five pathogens have established prevalence data from prior longitudinal studies in our study communities. No discrete outbreaks of these diseases were reported in the study communities during the sampling periods.

Leptospira spp.

Leptospirosis is caused by pathogenic *Leptospira* bacteria and one of the most common and widespread zoonoses worldwide. While *Leptospira* infections may be maintained in a diversity of wild and domestic animal reservoirs [1], rats are considered the primary source of human infection in urban habitats [2-4]. Infection typically occurs by vertical transmission or direct contact with either an animal reservoir or with environments that have been contaminated with the virus through urinary shedding [3,5,6]. The bacteria can enter a host via mucus membranes or broken skin and is thought to survive but not multiply in soils and freshwater bodies. As such, leptospirosis is primarily confined to tropic, developing parts of the world, where climatic and socioeconomic conditions favour both the endemicity of animal reservoirs and human exposure to the virus [2,4,7]. Leptospirosis outbreaks in humans generally occur during periods of flooding after heavy rainfall or following extreme climatic events such as hurricanes and monsoons [7,8].

Seoul orthohantavirus

Rats are the specific, natural hosts of the Seoul orthohantavirus (SEOV) [9], consistent with coevolution. Both humans and rats become infected via inhalation of excreted, air-borne viral particles or via wounding by infected rats [10,11]. In humans, SEOV infection can cause haemorrhagic fever with renal syndrome [12,13]. In rats, the infection is largely asymptomatic but has been linked to increased aggression in males from laboratory populations [14].

Toxoplasma gondii

The *Toxoplasma* genus comprises one species (*T. gondii*) of intracellular protozoan parasites with a two-stage life cycle that alternates between intermediate (almost all warm-blooded animals, including rats) and definitive hosts (felines) [15]. *T. gondii* occur as sexually mature parasites in the intestines of their definitive hosts and are excreted with their faeces as oocysts. When ingested by intermediate hosts, the oocysts then develop into tissue cysts throughout the host body. These cysts may persist as latent infections for many years before re-activating under immunocompromised conditions. Infection with *T. gondii*, a condition known as 'toxoplasmosis', is the most prevalent within warm and humid climates [16,17]. While toxoplasmosis is generally asymptomatic in humans, it may induce abortion and can cause severe brain and lung disease in individuals with suppressed immune systems, and infection prevalence in humans is >30% [15]. Rats are resistant to toxoplasmosis, making them an important source of human infection. Still, *T. gondii* can manipulate and inhibit the innate fear responses and mate choice of rat hosts, likely increasing their inter- and intraspecific transmission efficiency by depredation and sexual interactions respectively [18,19].

Angiostrongylus spp.

Angiostrongylus nematodes are thought to be transmitted to mammals via gastropod intermediate hosts. The parasites are typically ingested by terrestrial or freshwater snails and slugs via the infected faeces of their definitive hosts, developing within the gastropods to their third larval stage [20]. A definitive host becomes infected by feeding on infected gastropod tissue or mucus. Rats are the definitive hosts of *A. cantonensis*, or 'rat lungworm' [21]. Within the rat, *A. cantonensis* becomes blood-borne and undergo a final moult in the central nervous system before migrating to their definitive site in the pulmonary arteries. Infected rats are typically asymptomatic but can suffer brain damage that is manifested as cognitive and behavioural dysfunction under heavy infections [22]. Carrier hosts include crabs, freshwater shrimp, amphibians, flatworms, and fish [21]. In humans, the infection is considered an emerging zoonosis and is the causative agent of eosinophilic meningitis [23].

Capillaria spp.

Some nematodes in genus *Capillaria* require an intermediate host to complete their life cycle, while others are directly transmitted to final hosts. Animals shed the parasites as larvae or eggs in their faeces or by decomposition postmortem and generally become infected by ingestion of infected tissues or contaminated drinking water [24,25]. Out of roughly 300 known *Capillaria* species, four have been reported to infect humans. The best-studied example is *Capillaria hepatica*, which infects the liver of *Rattus* rats in its adult stage [26]. Its high prevalence and low pathogenicity in rats implies them as primary hosts for the parasite [25]. Infection appears to be endemic in rat populations worldwide but has been reported also in a suite of other mammals, including humans, and its ecology remains poorly understood [27].

Experimental design and study population

We collected Rattus norvegicus and R. rattus rats from seven slum communities in the city of Salvador-Bahía (692,819 km2) in northeastern Brazil (Figure 1): Pau da Lima [PdL], Alto do Cabrito [AdC], Marechal Rondon [MR], Sete de Abril [SdA], Nova Sussuarana [NS], Rio Sena [RS] and Nova Constituinte [NC]. Long-term cohort and cross-sectional studies within these communities have reported incidences of *Leptospira* spp., *Toxoplasma gondii*, Seoul orthohantavirus and zoonotic helminth infections in rats and humans for several decades [28-32]. As the communities of AdC and MR are adjacent and the rat sampling area across those two communities was contiguous, we pooled the data from MR and AdC. The areas sampled within communities ranged between 0.067 km^2 (RS) and 0.204 km^2 (NS) in size.

Topographically, Salvador is a city of hills and valleys. Altitudinal shifts in the city generally correspond to changes in the socioeconomic status of residents, with the lowest-income residents inhabiting the bottoms of the valleys. The residents of our study areas generally have a low socioeconomic status and live in poor-quality houses with insecure tenure. Sanitation is often inadequate, with untreated wastewater and sewerage flowing in open canals. The climate is equatorial and characterised by high humidity and high temperatures (annual range: 22-31°C), without marked seasons but with increased incidences of heavy rainfall during the wet season April-July. Prior studies have demonstrated that nearly 100% of rats trapped in the city of Salvador belong to the *R. norvegicus* species [33-35], which is generally more prevalent within urban slum environments.

The sampling was conducted through three longitudinal and cross-sectional rodent sampling projects between 2013 and 2023: sampling campaign A) 2016; B) 2021 – 2023; and C) 2013-

2018. Captures within campaign C (2013) were concentrated in June and July (n = 96 of 118 rats with recorded dates), coinciding with the onset and peak of Salvador's wet season (April–July). Campaign A (2016) captured predominantly in April and May (n = 39), also during the wet-season onset. Campaign B (2021–2022) captured in October and November (n = 21 rats with dates), during the dry season. Salvador's equatorial climate has no sharp seasonal boundaries, but leptospirosis risk typically peaks during the wet season due to flooding. Sampling campaign was included as a covariate in all infection and pharmaceutical detection models to account for systematic differences in infection prevalence or environmental pharmaceutical concentrations across periods.

During each sampling event, we placed two food-baited Tomahawk live-traps (45 × 16 × 16 cm3) within each of 30-40 domestic, peridomestic, and open backyards following a protocol previously described [36]. Sampling point selection was quasi-random, with a minimum distance of 15 meters separating each point. We placed traps within sampling points approximately 10 meters apart and aimed to maximise the efficiency of trapping by prioritising spots with a high chance of rat use (e.g. visible rat burrows, trash on the ground, permeable soil, unplastered walls) [35].

The traps were baited with variable food items (e.g. bread, sausages), inspected daily before noon and removed after four days. Captured rats were transported to a field lab and anaesthetised by ketamine or isoflurane inhalation prior to their weighing, bleeding by carotid puncture, euthanisation by thiopental overdose according to a standard protocol [37], and transportation to a laboratory for dissection. All rat trapping and handling followed protocols validated and approved by the Ethical Committee of the Animal Use (CEUA) protocols 003/2012, 019/2016, and Committee of Ethics in Research of the Institute of Collective Health – Federal University of Bahia (UFBA) – n°041/17, n° protocol 2.245.914.

We conducted environmental surveys (e.g. presence of open sewers, unpaved soil, trash) within a 20-meter radius of all traps, and pet ownership information was collected from households located within 20 meters of rat traps via resident questionnaires.

Captured and euthanised rats were double bagged and transported to a local laboratory for dissection and external assessment as per body condition (body mass and length), sex, pregnancy, age and species identity. Species identity was determined based on morphological characteristics and body measurements [38] and age was estimated using a scaled mass approach [39].

Sample storage and processing

We froze urine collected from the bladder and sera from blood samples at -70°C, and brain tissues at -20°C. Prior to Toxoplasma gondii screening, we extracted DNA from the urine using an automated Maxwell 16 System DNA Purification Kits (Promega Corp., Madison, WI). Tissue samples from the lungs, stomach and intestines were fixated at 50°C in formalin–acetic acid–alcohol (FAA) and stored at 4°C, as were faeces collected from the large intestine and stored in 10% formalin.

API analyses

A total of 97 pharmaceuticals were included in the liquid chromatography tandem mass spectrometry (LC-MS/MS) analysis (Table S1) together with 19 isotopically labelled internal

standards. We favoured brain tissues over other tissue samples as the subject of pharmaceutical screenings to optimise the detection of behaviour-modulating compounds that act via the central nervous system, and previous work in fish has shown that a range of environmental APIs bioconcentrate in brain tissues [40].

All the surrogate and internal standards used for positive identification and quantification of target compounds were classified as analytical grade (>98%). Purified water was prepared in-house using a Mili-Q Advantage system, including a UV radiation source (Millipore, Billerica, USA) and used as a mobile phase together with LC-MS/MS grade methanol (Merck, Darmstadt, Germany). LC-MS/MS grade formic acid (Sigma-Aldrich, Steinheim, Germany) was used to acidify the mobile chromatographic phases at 0.1%. HPLC grade acetonitrile was used as an extraction solvent.

Brain tissue samples (0.1 g) were pre-treated by adding 50 ng of each internal standard, 1.5 ml of acetonitrile, and extracted as described in detail previously [40]. In short, tissue samples underwent repeated (2x) solvent extraction, after which the collected supernatant was combined from both extraction rounds, evaporated to dryness, and reconstituted using 150 µL of LC-MS/MS grade methanol with 0.1% formic acid. Samples were then kept frozen at -18°C until analysis.

Samples were analysed using a triple stage quadrupole mass spectrometer (TSQ Quantiva, Thermo Scientific, San Jose, CA) equipped with a heated-electrospray ionization (HESI) ion source. The instrument was coupled to an Accela LC pump (Thermo Fisher Scientific, San Jose, CA) and a PAL HTC autosampler (CTC Analytics AG, Zwingen, Switzerland). A C18 phase Hypersil gold column (50 mm × 2.1 mm ID × 3 µm particles, Thermo Fisher Scientific, San Jose, CA, USA) with a guard column (2 mm, 2.1 mm, i.d. 5 µm particles) was used to separate the target analytes.

Two MS/MS transitions were used for positive identification of target compounds. Precision, limit of quantification (LOQ), and measurement of blank samples were used as the Quality assurance/Quality control for the analytical method used in this study, while recovery has been tested previously. Quantification of target compounds was carried out using the internal standard approach. Instrumental LOQs were derived from eight-point standard curve prepared in methanol and ranging from 0.01 to 50 ng/ml. The peak area corresponding to the lowest point in the standard curve that had a signal/noise ratio of >10 was then used for calculation of LOQs in individual samples (Table S1). Precision was expressed as a relative standard deviation (RSD) of response factors calculated for each point of the standard curve from the peak areas of the target analyte and its corresponding internal standard. The processing blanks were prepared following the same extraction protocol as tissue samples. We included processing blank samples in each analytical run and subtracted their pharmaceutical concentrations from those of test samples to control for background contamination. Quality assurance and control for the performance, repeatability, and recovery of the analytical method were documented during method development [41,42].

Additional details on the instrumental method including HESI ionizations, polarities, precursor/product ions, collision energies, and tube lens values are described elsewhere [42,43]. Additional details on brain tissue preparation and analysis, the pharmaceutical panel and the LOQs used can be found in the Supplementary Information (Table S1, Table S2).

Quantitative real-time polymerase chain reaction (qPCR) and kidney imprints for detection of Leptospira spp.

We isolated *Leptospira* cultures as previously described [44]. Whole rat kidney samples were homogenized in 5 ml of EMJH. We allowed the cell debris to settle for 10 minutes before using 0.5 ml of the cleared homogenate to inoculate 5 ml of fresh EMJH. The cultures were incubated at 29°C and monitored for growth under darkfield microscopy for up to 8 weeks.

We produced kidney imprints by pressing the longitudinally cut surface of a kidney onto a glass slide as described by Chagas-Junior, McBride [45]. The imprints were dried, acetone-fixed for 3 minutes, and incubated for 60 minutes with a primary rabbit polyclonal anti-leptospiral antibody (1:200 dilution) as previously described [46]. After rinsing with PBS, incubation with a goat anti-rabbit IgG-FITC conjugate (1:500 dilution), a second PBS wash, and drying, the slides were inspected for stained organisms using fluorescence microscopy, counting only intact spiral-shaped organisms.

We amplified *Leptospira* spp. DNA in rat urine using quantitative real-time PCR (qPCR) according to the methodology by Costa, Wunder [37]. Briefly, DNA was extracted from 200μL of previously frozen urine using the Maxwell 16 System DNA Purification Kit. *Leptospira* detection was carried out using qPCR targeting the *lipL32* gene, which is specific to pathogenic *Leptospira* species. The qPCR assay used an eight-point calibration curve based on quantified genomic DNA from the L1-130 strain.

Reactions were performed in duplicate on an ABI 7500 Real-Time PCR System with a TaqMan probe. The qPCR reaction was performed in a final volume of 25 µL containing 12.5 µL of Platinum Quantitative PCR SuperMix-UDG (Invitrogen), 500 nM of each primer, 100 nM probe, 5 µL of DNA template, and ultrapure water. Cycling conditions were 2 min at 50 °C, 10 min at 95 °C, followed by 45 cycles of 95 °C for 15 s and 60 °C for 1 min [47].

Each run included a no-template control (NTC), which consistently showed no amplification or Ct values > 40, indicating absence of contamination or non-specific fluorescence. PCR inhibition and extraction efficiency were monitored using primers for the rodent housekeeping gene GADPH, confirming adequate DNA quality and absence of significant inhibition. Samples with amplification within 40 cycles were considered positive and confirmed by Sanger sequencing.

In the 83 animals for which both urine qPCR and kidney immunofluorescence imprint data were available concurrently, infection status was concordant in 77.1% of cases (Cohen's $\kappa = 0.37$). Discordant cases were predominantly animals classified as positive by urine qPCR but negative by kidney imprint ($n = 17$), a pattern consistent with the known biology of leptospiral infection: urinary shedding detectable by molecular methods can precede or occur in the absence of kidney tissue colonisation at the level detectable by immunofluorescence. Only 2 animals were imprint-positive but urine qPCR-negative. This directional pattern confirms the higher sensitivity of PCR-based detection and supports our decision to use qPCR as the primary infection assignment, with kidney imprints used only when qPCR data were unavailable.

Enzyme-linked immunosorbent assay (ELISA) for detection of Seoul orthohantavirus (SEOV)

We determined SEOV positivity using a modified enzyme-linked immunosorbent assay (ELISA) based on the Andes hantavirus recombinant nucleoprotein antigen, as described by Padula et al. (2000).[(24)] Briefly, thawed sera samples were diluted 1:400 in phosphate-buffered saline (PBS) at pH 7.4. A purified recombinant Andes hantavirus nucleoprotein (N-ANDV), produced in *Escherichia coli*, was used as the specific antigen at 2 µg/mL per well in microwell plates. Peroxidase-conjugated IgG antibodies (anti-Rat and anti-*Peromyscus leucopus*) were used as the secondary conjugate. Antigen–antibody binding was detected by colorimetric reaction and measured as optical density (OD). Final OD values were obtained by subtracting the OD of wells coated with a recombinant negative-sense-encoding nucleoprotein (background control). Sera were assigned SEOV-positive when corrected ODs exceeded the positivity cutoff at a 1:400 dilution. Reagents were supplied by Kirkegaard & Perry Laboratories (Gaithersburg, MD, USA). No negative serum control samples showed OD values above the positivity threshold.

Polymerase chain reaction (PCR) for detection of Toxoplasma gondii

We detected *Toxoplasma gondii* DNA in rat urine using a single-tube nested PCR targeting the ITS1 (18S–5.8S ribosomal RNA intergenic spacer) region. DNA was extracted from 200 µL of previously frozen urine samples using phenol-chloroform-isoamyl alcohol (Sigma-Aldrich). Two successive amplifications were performed in a single closed tube using 18S-5.8S ITS1-targeted primers. PCR conditions, cycling parameters, and controls followed established protocols. Sensitivity was evaluated using serial dilutions of *T. gondii* genomic DNA in both clean and urine-spiked backgrounds. Specificity was evaluated in silico by BLAST searching against GenBank sequences from closely related protozoa, and empirically by testing against DNA extracts from phylogenetically related organisms. Each run included a no-template negative control (NTC); all NTCs yielded no amplification product, confirming the absence of contamination.

Helminth egg and larvae detection

We determined infections by *Angiostrongylus* spp. and *Capillaria* spp. using a modified Hoffman sedimentation-concentration technique. Briefly, 1 g of formalin-fixed faeces was suspended in water, homogenised, and filtered. The filtered suspension was allowed to sediment in a conical vessel, and aliquots were collected from the sediment tip using a pipette. Aliquots were examined by light microscopy for helminth eggs, with species identification guided by helminth lists previously described for *Rattus norvegicus*. Helminth identification in faeces was confirmed by detection of adult parasites in formalin–acetic acid–alcohol (FAA)-fixated samples of stomach, intestine, heart, and lung tissue.

Statistical analyses

We conducted all statistical analyses in R v.4.2.3 R Core[48], applying a standard $p < 0.05$ significance threshold. We tested for co-occurrence of the six most frequently detected compounds (MFD) using pairwise chi-square tests of independence on binary detection data.

To characterise associations between API detection and infection status in rats, we used generalised linear models (GLM) with a binomial error distribution. We selected all non-

pharmaceutical predictors on a pathogen-to-pathogen basis. The response variables were binary infection status for *Leptospira* spp., SEOV, *Toxoplasma gondii*, *Capillaria* spp., *Angiostrongylus* spp., and *Capillaria* and *Angiostrongylus* spp. combined. We fitted two models to each response variable: one testing for associations with the detection of any API and another testing for associations with the detection of each of the six most frequently detected APIs. All final models were determined by two successive rounds of multivariate model selection: the selection of explanatory variables indicated by previous literature [32,49,50], followed by the selection of pharmaceutical focal predictors. The first round of multivariate model selection included predictors with $p \geq 0.2$ in bivariate linear models (Table S4) with a binomial error distribution and was conducted using the dredge function in R package MuMIn [33,34]. In both rounds, we selected best models based on AICc criteria (Table S5-S19). If multiple models were closely ranked (ΔAIC < 2), we selected the most parsimonious model. We evaluated final models for multicollinearity using the variance inflation factor (VIF) and removed variables with a VIF >10. No variables in the remaining models had a VIF>4 (Table S20).

To identify environmental factors associated with pharmaceutical detection in the rats, we used generalised linear models (GLM) with a binomial error distribution. The response variables were detection of any API, regardless of identity, and detection of each of the six most frequently detected APIs. We selected a panel of 25 potential predictors that reflect environmental quality based on previous literature [51,52] and are potential sources of API exposure and contamination (e.g. trash, open sewers), testing their association with each response variable using bivariate linear models with a binomial error distribution. Applying a similar modelling approach as for the detection of associations between API detection and infection, we limited the multivariate model selection to predictors with $p \geq 0.2$ in bivariate models (Table S21) and selected the final models based on AICc criteria (Table S22-28). We removed variables with a VIF >10 (Table S29).

To assess whether systematic segregation of API-exposed and unexposed rats across the study area could drive the observed associations, we examined the spatial distribution of infection status and pharmaceutical detection relative to trap locations. In communities where georeferenced trap data and *Leptospira* infection status were both available (Alto do Cabrito, Rio Sena, and Nova Constituinte; n = 110 rats at 61 trap locations), *Leptospira*-positive and -negative rats were captured at identical trap coordinates in 13 of 61 trap locations (21%). In Pau da Lima, which contributed most of pharmaceutical detection data, the total trapping area spanned approximately 920 m × 700 m, with all pharmaceutical exposure categories (API-positive and API-negative) represented throughout the area. Rat home ranges in this system have been estimated at 20–50 m [34], meaning that API-positive and API-negative animals systematically occupied overlapping home ranges. These observations are inconsistent with habitat-use segregation as an explanation for the observed API–infection associations and support the interpretation that the associations reflect pharmacological effects rather than spatial confounding.

Table S1.

Mean limit of quantification (LOQ) for all target pharmaceuticals included in the analysis of rat brain tissues, representing their average LOQ value across all samples (n = 180).

**Compound**	**Mean LOQ (ng g^{-1})**
Alfuzosin	0.432
Alprazolam	0.136
Amiodiarone	0.085
Amitryptiline	0.079
Atenolol	15.077
Atorvastatin	0.082
Atracurium	0.113
Azelastine	0.086
Azithromycin	0.063
Beclomethazone	2.664
Benzoylecgonine	0.766
Bisoprolol	0.092
Bromocriptin	7.739
Budesonide	8.121
Buprenorphin	0.105
Bupropion	0.490
Caffeine	9.676
Carbamazepin	0.129
Chloprothixen	0.086
Chlorpromazine	0.079
Cilazapril	0.100
Citalopram	0.067
Clarithromycine	0.111
Clemastine	0.049
Clindamycin	0.085
Clomipramine	0.075
Clonazepam	0.484
Clotrimazol	0.443
Cocaine	7.916
Codeine	1.445
Cyproheptadine	0.132
Desloratidin	0.393
Diclofenac	1.112
Dicycloverin	0.043

Dihydroergotamin	0.520
Diltiazem	0.089
Diphenhydramin	0.097
Dipyridamol	0.138
Donepezil	0.073
Duloxetin	0.503
Eprosartan	0.508
Erythromycine	5.483
Etonorgestrel	3.858
Ezetimibe	3.849
Felodipine	0.594
Fenofibrate	0.126
Fexofenadine	0.090
Finasteride	1.634
Flecainide	0.090
Fluconazole	0.262
flunitrazepam	0.039
Fluoxetin	0.068
Flupentixol	0.101
Fluphenazine	0.075
Flutamid	0.395
Glibenclamide	0.327
Glimepiride	0.120
Haloperidol	0.117
Hydroxyzine	0.075
Ibersartan	0.055
Ketoconazole	0.500
Levomepromazine	0.077
Loperamide	0.087
Maprotilin	0.086
Meclozine	0.042
Memantin	0.409
Metoprolol	0.321
Mianserin	0.137
Miconazole	0.554
Mirtazapine	0.051
Naloxon	1.231
Nefazodon	0.091
Orphenadrin	0.091
Oxazepam	0.188

Paracetamol	10.649
Paroxetin	0.084
Perphenazine	0.067
Pizotifen	0.094
Promethazin	0.064
Propranolol	0.573
Ranitidine	0.977
Repaglinide	0.074
Risperidone	0.026
Rosuvastatin	1.649
Roxithromycine	0.101
Sertraline	0.402
Sotalol	60.431
Sulfamethoxazol	1.167
Tamoxifen	0.078
Telmisartan	0.092
Terbutalin	5.154
Tramadol	0.171
Trihexyphenidyl	0.092
Trimetoprim	0.632
Venlavafaxin	0.078
Verapamil	0.100
Zolpidem	0.106

Table S2.

Pairwise chi-square tests examining the co-occurrence of six active pharmaceutical ingredients in rat brain tissues (n = 152).

**Drug class 1**	**Drug class 2**	**ChiSq**	**p(ChiSq)**
Citalopram	Donepezil	0.456	0.500
Citalopram	Azithromycin	0.569	0.451
Citalopram	Clindamycin	0.011	0.918
Citalopram	Caffeine	0.000	1.000
Citalopram	Haloperidol	0.106	0.745
Donepezil	Azithromycin	1.481	0.224
Donepezil	Clindamycin	0.000	1.000
Donepezil	Caffeine	0.000	1.000
Donepezil	Haloperidol	0.000	1.000
Azithromycin	Clindamycin	0.000	1.000
Azithromycin	Caffeine	0.636	0.425
Azithromycine	Haloperidol	0.919	0.338
Clindamycin	Caffeine	0.000	1.000
Clindamycin	Haloperidol	0.000	1.000
Caffeine	Haloperidol	0.002	0.968

Table S3.

Final binomial regression (GLM) model testing for the effect of API detection on Leptospira spp. infection risk, when azithromycin detections are excluded (point = odds ratio (OR) estimate, line = 95% confidence interval (CI), * = $p < 0.10$, ** = $p < 0.05$, *** = $p < 0.01$, N **= 152).**

**Predictor of infection**	**OR (95% CI)**	**p(ChiSq)**
Age (weeks)	1.241 (1.076 – 1.432)	0.003 ***
Azithromycin detection	0.366 (0.141 – 0.948)	0.038 **

Table S4.

Bivariate GLM outcomes between each of five binary infection response variables (LEPTO = *Leptospira* spp., SEOV = Seoul Hantavirus, TOXO = *Toxoplasma gondii*, CAPI = *Capillaria* spp., ANGIO = *Angiostrongylus* spp., C + A = *Capillaria* and *Angiostrongylus* spp. detections) and 26 physiological, environmental and logistic predictors (empty cell = term excluded from bivariate analyses).

	**P (>z) against each infection response**					
**Term**	**LEPTO**	**SEOV**	**TOXO**	**CAPI**	**ANGI**	**C + A**
Rat properties						
Age	0.064	0.010	0.620	0.814	0.135	0.093
Wounding	0.109	0.190	0.996			
Sex	0.357	0.029	0.994	0.127	0.282	0.334
Body condition	0.291	0.935	0.167	0.013	0.075	0.266
Pregnancy	0.123		0.238			
Environmental features						
Open sewers	0.728	0.273	0.482	0.105	0.996	0.994
Steep ground incline	0.906	0.143	0.573	0.429	0.373	0.858
Trash	0.928	0.120	0.258	0.281	0.708	0.355
Rat accessible food	0.353	0.773	0.634	0.272	0.846	0.514
Trees	0.277	0.141	0.925	0.688	0.417	0.708
Paved ground	0.990	0.274	0.968	0.039	0.708	1.000
Unpaved ground	0.277	0.124	0.776	0.021		
Any pets	0.261	0.585	0.828	0.594	0.159	0.106
Mammal pets	0.459	0.699	0.658	0.486	0.327	0.307
Cats	0.374		0.993			
Number of cats	0.239		0.996			
Avian pets					0.995	
Chicken					0.997	
Standing water	0.658	0.717	0.298	0.439	0.565	0.908
Residential area	0.988	0.992	0.995	0.989	0.996	0.995
Rat capture event						
Date			0.796	0.356	0.353	0.167
Rat sampling project						
MRC1			0.459	0.000		
Otímo	0.988		0.565			
Community						
NC	0.183	0.135	0.745	0.802		
NS			0.375			
PdL	0.991		0.557	0.000		
RS	0.395	0.853	0.995	0.219		
SdA	0.991		0.998			

Table S5

Best models explaining *Leptospira* infection in rats, excluding pharmaceutical predictors (x = term included in model; n = 100). Each row corresponds to one model.

Model terms		df	AIC
Rat age	Community		
x		2	120.9

Table S6.

Best models explaining *Leptospira* infection in rats, including API detection as a predictor (x = term included in model; n = 100). Each row corresponds to one model.

Model terms		df	AIC
Rat age	API		
x	x	3	115

Table S7.

Best models explaining *Leptospira* infection in rats, including the detection of six APIs as predictors x = term included in model; n = 100). Each row corresponds to one model.

Model terms							df	AIC
**Rat age**	**AZI**	**CAF**	**CIT**	**CLI**	**DON**	**HAL**		
**x**	**x**	x	x		x		6	113.9
x	x	x	x				5	114.0
x	x	x					4	114.4
x	x	x			x		5	114.9
x	x	x	x			x	6	115.2
x	x	x	x		x	x	7	115.3
x	x	x				x	5	115.5
x	x	x	x	x	x		7	115.8

Table S8.

Best models explaining SEOV infection in rats, excluding pharmaceutical predictors (x = term included in model; n = 85). Each row corresponds to one model.

Model terms								
**Trash**	**Community**	**Trees**	**Rat age**	**Sex**	**Unpaved soil**	**Wounding**	**df**	**AIC**
x			x	x			4	91.829
x			x	x	x		5	92.177
			x	x	x		4	92.611
x		x	x	x			5	92.970
		x	x	x	x		5	93.180
x		x	x	x	x		6	93.199
	x		x	x	x		6	93.217
			x	x			3	93.469
x	x		x	x			6	93.575
x			x	x		x	5	93.733
	x		x	x			5	93.806

Table S9.

Best models explaining SEOV infection in rats, including API detection as a predictor (x = term included in model; n = 85). Each row corresponds to one model.

Model terms			df	AIC
API	Age	Sex		
	x	x	3	94.056
x	x	x	4	96.054

Table S10.

Best models explaining SEOV infection in rats, including the detection of six APIs as predictors (CIT = Citalopram, DON = Donepezil, AZI = Azithromycine, CLI = Clindamycine, CAF = Caffeine and HAL = Haloperidol, x = term included in model; n = 85). Each row corresponds to one model.

Model terms									
**AZI**	**CAF**	**CIT**	**CLI**	**DON**	**Age**	**HAL**	**Sex**	**df**	**AIC**
		x			x		x	4	93.565
					x		x	3	94.056
		x			x	x	x	5	94.177
		x	x		x		x	5	94.241
					x	x	x	4	94.427
		x		x	x		x	5	94.710
		x	x		x	x	x	6	94.865
	x	x			x		x	5	95.075
			x		x		x	4	95.100
		x		x	x	x	x	6	95.141
				x	x		x	4	95.178
		x	x	x	x		x	6	95.281
				x	x	x	x	5	95.341
x		x			x		x	5	95.416
			x		x	x	x	5	95.460
	x				x		x	4	95.491

Table S11.

Best models explaining *Toxoplasma* infection in rats, excluding pharmaceutical predictors x = term included in model; n = 148). Each row corresponds to one model.

Model term		
Body condition	df	AIC
	1	43.98604
x	2	44.05162

Table S12.

Best models explaining *Toxoplasma* infection in rats, including API detection as a predictor (x = term included in model; n = 148). Each row corresponds to one model.

Model term	df	AIC
API		
x	2	58.218
	1	58.381

Table S13.
Best models explaining *Toxoplasma* infection in rats, including the detection of six APIs as predictors (CIT = Citalopram, DON = Donepezil, AZI = Azithromycine, CLI = Clindamycine, CAF = Caffeine and HAL = Haloperidol, x = term included in model; n = 148). Each row corresponds to one model.

Model terms							
**AZI**	**CAF**	**CIT**	**CLI**	**DON**	**HAL**	**df**	**AIC**
				x		2	58.183
						1	58.382
x				x		3	58.498
x						2	58.955
	x			x		3	59.245
			x	x		3	59.245
				x	x	3	59.366
	x					2	59.481
			x			2	59.481
x			x	x		4	59.573
					x	2	59,584
x	x			x		4	59,709
x				x	x	4	59,843
x			x			3	60,069
		x		x		3	60,179

Table S14.

Best models explaining *Capillaria* infection in rats, excluding pharmaceutical predictors x = term included in model; n = 124). Each row corresponds to one model.

**Model terms**							
**Area**	**Paved soil**	**Rat trapping project**	**Sex**	**Body condition**	**Unpaved soil**	**df**	**AIC**
		x				2	135.837
		x	x			3	137.041
		x		x		3	137.297
	x	x				3	137.457
		x			x	3	137.659
x						4	137.722
x		x				4	137.722

Table S15.

Best models explaining *Capillaria* infection in rats, including API detection as a predictor (x = term included in model; n = 124). Each row corresponds to one model.

**Model terms**			
**API**	**Rat trapping project**	**df**	**AIC**
	x	2	145.044
x	x	3	146.957

Table S16.

Best models explaining *Capillaria* infection in rats, including the detection of six APIs as predictors (CIT = Citalopram, DON = Donepezil, AZI = Azithromycine, CLI = Clindamycine, CAF = Caffeine and HAL = Haloperidol, x = term included in model; n = 148). Each row corresponds to one model.

**Model terms**							**df**	**AIC**
**AZI**	**CAF**	**CIT**	**CLI**	**DON**	**HAL**	**Rat trapping project**		
	x	x				x	4	141.274
		x				x	3	141.737
	x	x		x		x	5	142.414
		x		x		x	4	142.857
x	x	x				x	5	142.894
x		x				x	4	143.004
	x	x			x	x	5	143.236
	x	x	x			x	5	143.271

Table S17.

Best models explaining *Angiostrongylus* infection in rats, excluding pharmaceutical predictors (x = term included in model; n = 33). Each row corresponds to one model.

Model terms				
**Any pet**	**Rat age**	**Body condition**	**df**	**AIC**
x		x	3	19.459
		x	2	19.538
x			2	20.937
			1	21.067
x	x	x	4	21.119
	x	x	3	21.416
	x		2	21.432

Table S18.
Best models explaining *Angiostrongylus* infection in rats, including API detection as a predictor (x = term included in model; n = 33). Each row corresponds to one model.

Model term	df	AIC
API		
	x	26.375
x	x	27.048

Table S19.

Best models explaining *Angiostrongylus* infection in rats, including the detection of six APIs as predictors (CIT = Citalopram, DON = Donepezil, AZI = Azithromycine, CLI = Clindamycine, CAF = Caffeine and HAL = Haloperidol, x = term included in model; n = 33). Each row corresponds to one model.

Model terms							
**AZI**	**CAF**	**CIT**	**CLI**	**DON**	**HAL**	**df**	**AIC**
			x			2	25.139
	x		x			3	25.955
						1	26.375
			x		x	3	26.498
			x	x		3	26.559
x			x			3	26.778
		x	x			3	27.095

Table S20.

Best models explaining *Capillaria* and *Angiostrongylus* infection in rats, excluding pharmaceutical predictors (x = term included in model; n = 33). Each row corresponds to one model.

Model terms				df	AIC
Pets	Date	Rat age	Body condition		
	x		x	3	17.8
	x	x	x	4	18.8
x	x		x	4	19.5

Table S21.

Best models explaining *Capillaria* and *Angiostrongylus* infection in rats, including API detection as a predictor (x = term included in model; n = 33). Each row corresponds to one model.

Model terms			df	AIC
API	Date	Body condition		
	x	x	3	23.1
x	x	x	4	24.7

Table S22.

Best models explaining *Capillaria* and *Angiostrongylus* infection in rats, including the detection of six APIs as predictors (CIT = Citalopram, DON = Donepezil, AZI = Azithromycine, CLI = Clindamycine, CAF = Caffeine and HAL = Haloperidol, x = term included in model; n = 33). Each row corresponds to one model.

**Model terms**									
**AZI**	**CAF**	**CIT**	**CLI**	**DON**	**HAL**	**Date**	**Body condition**	**df**	**AIC**
						x	x	3	23.1
x						x	x	4	23.5
		x				x	x	4	23.6
					x	x	x	4	23.9
	x					x	x	4	24.1
		x			x	x	x	5	24.2
x		x				x	x	5	24.4
			x			x	x	4	24.9
	x				x	x	x	5	24.9
x	x					x	x	5	24.9
x					x	x	x	5	25.1
				x	x	x	x	4	25.1

Table S23.
Variance inflation factors of all predictors in final logistic regression models describing infection risk with >1 predictor.

**Model response**		**Term**	**VIF**
Infection risk:	*Leptospira*	Rat age	1.157
		API detection	1.157
	Leptospira	Rat age	1.187
		Citalopram	1.192
		Donepezil	1.069
		Azithromycine	1.037
		Caffeine	1.000
	SEOV	Rat age	1.044
		Sex	1.030
		API detection	1.026
	SEOV	Rat age	1.031
		Sex	1.052
		Citalopram	1.026
	Capillaria	Rat trapping project	1.038
		Citalopram	1.037
		Caffeine	1.004

Table S24.

Bivariate GLM outcomes between each of 11 binary response variables (binary detection of any API (ALL) and of six compounds (CIT = Citalopram, DON = Donepezil, AZI = Azithromycine, CLI = Clindamycine, CAF = Caffeine and HAL = Haloperidol) and 26 physiological, environmental and logistic predictors.

	**p (>z) against each pharmaceutical detection response**						
**Term**	**ALL**	**CIT**	**DON**	**AZI**	**CLI**	**CAF**	**HAL**
Rat properties							
Age	0.222	0.077	0.254	0.281	0.858	0.304	0.386
Wounding	0.197	0.688	0.427	0.915	0.994	0.774	0.998
Sex	0.476	0.300	0.487	0.340	0.514	0.378	0.197
Body condition	0.994	0.840	0.924	0.821	0.053	0.693	0.818
Environmental features							
Open sewers	0.570	0.749	0.930	0.770	0.371	0.888	0.999
Steep ground incline	0.981	0.135	0.252	0.905	0.707	0.859	0.994
Fence	0.451	0.571	0.601	0.134	0.479	0.988	0.327
Trash	0.710	0.467	0.514	0.031	0.610	0.368	0.232
Rat accessible food	0.796	0.408	0.907	0.069	0.182	0.942	0.493
Construction material	0.886	0.984	0.764	0.891	0.058	0.993	0.852
Trees	0.143	0.327	0.633	0.001	0.571	0.571	0.679
Paved ground	0.327	0.714	0.273	0.900	0.088	0.634	0.808
Unpaved ground	0.185	0.055	0.116	0.647	0.758	0.244	0.600
Any pets	0.654	0.191	0.546	0.584	0.780	0.242	0.993
Mammal pets	0.588	0.776	0.750	0.211	0.347	0.841	0.201
Avian pets	0.407	0.296	0.358	0.466	0.993	0.463	0.066
Chicken	0.539	0.962	0.133	0.908	0.992	0.991	0.774
Standing water	0.984	0.745	0.388	0.795	0.323	0.807	0.327
Residential area	0.925	0.298	0.992	0.992	0.371	0.371	0.396
Public area	0.865	0.476	0.393	0.992	0.920	0.920	0.312
Open area	0.488	0.769	0.989	0.993	0.221	0.221	0.182
Rat capture event							
Date	0.991	0.370	0.833	0.165	0.148	0.819	0.993
Rat sampling project							
MRC1	0.441	0.840	0.205	0.786	0.045	0.491	0.443
Otímo	0.309	0.054	0.991	0.991	0.991	0.293	0.024
Community							
NC	0.095	0.135	0.122	0.303	0.479	0.995	0.710
NS	0.517	0.023	0.991	0.991	0.996	0.389	0.996
PdL	0.182	0.112	0.029	0.632	0.368	0.104	0.008
RS	0.969	0.014	0.274	0.955	0.995	0.997	0.165
SdA	0.900	0.355	0.994	0.995	0.998	0.999	0.997
API extraction details							
Date	0.193	0.103	0.758	0.143	0.054	0.248	0.999

Table S25.

Best models explaining API detection in rats (x = term included in model; n = 148). Each row corresponds to one model.

**Model terms**					
**Area**	**Trees**	**API extraction date**	**Unpaved soil**	**df**	**AIC**
				1	204.252
	x		x	3	204.377
	x			2	204.416
			x	2	204.427
		x		2	204.682
	x	x		3	204.962
		x	x	3	205.260
	x	x	x	4	205.343

Table S26.

Best models explaining citalopram detection in rats (x = term included in model; n = 148). Each row corresponds to one model.

Model terms							
**Any pets**	**Community**	**Rat age**	**Tissue extraction date**	**Rat sampling project**	**Unpaved soil**	**df**	**AIC**
	x	x	x		x	7	127.603
	x	x			x	6	128.262
		x			x	3	128.615
x	x	x	x		x	8	129.204
	x	x		x	x	7	129.377
	x		x		x	6	129.595
	x	x	x	x	x	8	129.598

Table S27.

Best models explaining donepezil detection in rats (x = term included in model; n = 148). Each row corresponds to one model.

Model terms			df	AIC
Community	Chicken	Unpaved soil		
x	x	x	8	115.562
x		x	7	116.319

Table S28.

Best models explaining azithromycin detection in rats (x = term included in model; n = 148). Each row corresponds to one model.

**Model terms**						
**Trash**	**Human food**	**Trees**	**API extraction date**	**Fence**	**df**	**AIC**
x		x	x	x	5	82.552
x		x		x	4	83.071
x		x	x		4	83.154
		x	x	x	4	84.063
		x		x	3	84.318
x		x			3	84.443
x	x	x	x	x	6	84.536

Table S29.

Best models explaining clindamycin detection in rats (x = term included in model; n = 148). Each row corresponds to one model.

Model terms							
**Human food**	**Brain tissue extraction date**	**Building materials**	**Paved ground**	**Rat sampling project**	**Body condition**	**df**	**AIC**
		x			x	3	62.934
x		x			x	4	63.341
		x		x		3	63.453
		x				2	64.033
		x	x		x	4	64.079
	x	x				3	64.135
x		x				3	64.239
		x	x			3	64.260
x		x		x		4	64.262
x		x	x			4	64.421
		x		x	x	4	64.454
x		x	x		x	5	64.552
	x	x			x	4	64.600
					x	2	64.698
				x		2	64.889

Table S30.

Best models explaining caffeine detection in rats (x = term included in model; n = 148). Each row corresponds to one model.

Model terms			
Community	Rat wounds	df	AIC
x		4	52.705
x	x	5	52.957

Table S31.

Best models explaining haloperidol detection in rats (x = term included in model; n = 148). Each row corresponds to one model.

**Model terms**					
**Area**	**Any mammal pet**	**Open area**	**Rat sampling project**	**df**	**AIC**
			x	3	56.659
	x		x	4	56.835
+			x	7	57.187
+	x		x	8	57.328
+				6	57.336
		x	x	4	57.960
	x	x	x	5	58.290
+	x			7	58.403

Table S32.

Variance inflation factors of all predictors in final logistic regression models describing API detection with >1 predictor.

**Model response**		**Term**	**VIF**
API detection:	Citalopram	Unpaved soil	1.070
		Rat age	1.070
	Azithromycin	Fence	1.032
		Trees	1.032

Data S1. (separate file)

Raw data describing the outcomes of pharmaceutical screenings of rat brain tissues.

Data S2. (separate file)

Raw data describing the outcomes of physiological and demographic assessments of rats, and the of environmental surveys at their trapping locations.

References

[1] Andersen-Ranberg EU, Pipper C, Jensen PM. Global patterns of Leptospira prevalence in vertebrate reservoir hosts. Journal of wildlife diseases. 2016;52(3):468-477.

[2] Reis RB, Ribeiro GS, Felzemburgh RD, et al. Impact of environment and social gradient on Leptospira infection in urban slums. PLoS neglected tropical diseases. 2008;2(4):e228.

[3] Haake DA, Levett PN. Leptospirosis in humans. Leptospira and leptospirosis. 2015:65-97.

[4] Boey K, Shiokawa K, Rajeev S. Leptospira infection in rats: A literature review of global prevalence and distribution. PLoS Neglected Tropical Diseases: Public Library of Science; 2019.

[5] Picardeau M. Virulence of the zoonotic agent of leptospirosis: still terra incognita? Nature Reviews Microbiology. 2017;15(5):297-307.

[6] Samrot AV, Sean TC, Bhavya KS, et al. Leptospiral infection, pathogenesis and its diagnosis—A review. Pathogens. 2021;10(2):145.

[7] Pappas G, Papadimitriou P, Siozopoulou V, et al. The globalization of leptospirosis: worldwide incidence trends. International journal of infectious diseases. 2008;12(4):351-357.

[8] Cunha DL, Mendes MP, Marques M. Environmental risk assessment of psychoactive drugs in the aquatic environment. Environmental Science and Pollution Research: Springer Verlag; 2019. p. 78-90.

[9] Plyusnin A, Vapalahti O, Vaheri A. Hantaviruses: genome structure, expression and evolution. Journal of General Virology. 1996;77(11):2677-2687.

[10] Glass G, Childs J, Korch G, et al. Association of intraspecific wounding with hantaviral infection in wild rats (Rattus norvegicus). Epidemiology & Infection. 1988;101(2):459-472.

[11] Nuzum EO, Rossi CA, Stephenson EH, et al. Aerosol transmission of Hantaan and related viruses to laboratory rats. The American journal of tropical medicine and hygiene. 1988;38(3):636-640.

[12] Lee HW, Lee PW, Johnson KM. Isolation of the etiologic agent of Korean hemorrhagic fever. Journal of infectious diseases. 1978;137(3):298-308.

[13] Nichol ST, Spiropoulou CF, Morzunov S, et al. Genetic identification of a hantavirus associated with an outbreak of acute respiratory illness. Science. 1993;262(5135):914-917.

[14] Klein SL, Bird BH, Nelson RJ, et al. Environmental and physiological factors associated with Seoul virus infection among urban populations of Norway rats. Journal of Mammalogy. 2002;83(2):478-488.

[15] Saadatnia G, Golkar M. A review on human toxoplasmosis. Scandinavian journal of infectious diseases. 2012;44(11):805-814.

[16] Tenter AM, Heckeroth AR, Weiss LM. Toxoplasma gondii: from animals to humans. International journal for parasitology. 2000;30(12-13):1217-1258.

[17] Bojar I, Szymanska J. Environmental exposure of pregnant women to infection with Toxoplasma gondii-state of the art. Annals of Agricultural and Environmental Medicine. 2010;17(2):209-214.

[18] Terpsidis KI, Papazahariadou MG, Taitzoglou IA, et al. Toxoplasma gondii: reproductive parameters in experimentally infected male rats. Experimental parasitology. 2009;121(3):238-241.

[19] Vyas A. Parasite-augmented mate choice and reduction in innate fear in rats infected by Toxoplasma gondii. Journal of Experimental Biology. 2013;216(1):120-126.

[20] Mackerras MJ, Sandars DF. The life history of the rat lung-worm, Angiostrongylus cantonensis (Chen)(Nematoda: Metastrongylidae). Australian Journal of Zoology. 1955;3(1):1-21.

[21] Alicata JE. Biology and distribution of the rat lungworm, Angiostrongylus cantonensis, and its relationship to eosinophilic meningoencephalitis and other neurological disorders of man and animals. Advances in parasitology. 1965;3:223-248.

[22] Luo S, OuYang L, Wei J, et al. Neuronal apoptosis: Pathological basis of behavioral dysfunctions induced by Angiostrongylus cantonensis in rodents model. Korean Journal of Parasitology. 2017;55(3):267-278.

[23] Morassutti AL, Thiengo SC, Fernandez M, et al. Eosinophilic meningitis caused by Angiostrongylus cantonensis: an emergent disease in Brazil. Memórias do Instituto Oswaldo Cruz. 2014;109:399-407.

[24] Cross JH, Banzon T, Clarke MD, et al. Studies on the experimental transmission of Capillaria philippinensis in monkeys. Transactions of the Royal society of Tropical Medicine and Hygiene. 1972;66(6):819-827.

[25] Fuehrer H-P. An overview of the host spectrum and distribution of Calodium hepaticum (syn. Capillaria hepatica): part 1—Muroidea. Parasitology Research. 2014;113:619-640.

[26] Ceruti R, Sonzogni O, Origgi F, et al. Capillaria hepatica infection in wild brown rats (Rattus norvegicus) from the urban area of Milan, Italy. Journal of Veterinary Medicine, Series B. 2001;48(3):235-240.

[27] Fuehrer H-P, Igel P, Auer H. Capillaria hepatica in man-an overview of hepatic capillariosis and spurious infections. Parasitology Research. 2011(109):969-979.

[28] Carvalho-Pereira T, Souza FN, Santos LRN, et al. The helminth community of a population of Rattus norvegicus from an urban Brazilian slum and the threat of zoonotic diseases. Parasitology. 2018;145(6):797-806.

[29] Costa F, Porter FH, Rodrigues G, et al. Infections by Leptospira interrogans, seoul virus, and bartonella spp. among norway rats (rattus norvegicus) from the Urban slum environment in Brazil. Vector-Borne and Zoonotic Diseases. 2014;14(1):33-40.

[30] Costa F, Hagan JE, Calcagno J, et al. Global morbidity and mortality of leptospirosis: a systematic review. PLoS neglected tropical diseases. 2015;9(9):e0003898.

[31] Dattoli VCC, Veiga RV, Cunha SS, et al. Oocyst ingestion as an important transmission route of toxoplasma gondii in Brazilian Urban children. Journal of Parasitology. 2011;97(6):1080-1084.

[32] Souza FN, Santos MA, Alves DA, et al. Angiostrongylus cantonensis in urban populations of terrestrial gastropods and rats in an impoverished region of Brazil. Parasitology. 2021;148(8):994-1002.

[33] Faria MTd, Calderwood MS, Athanazio DA, et al. Carriage of Leptospira interrogans among domestic rats from an urban setting highly endemic for leptospirosis in Brazil. Acta Tropica. 2008;108(1):1-5.

[34] Kajdacsi B, Costa F, Hyseni C, et al. Urban population genetics of slum-dwelling rats (Rattus norvegicus) in Salvador, Brazil. Molecular ecology. 2013;22(20):5056-5070.

[35] Costa F, Ribeiro GS, Felzemburgh RDM, et al. Influence of Household Rat Infestation on Leptospira Transmission in the Urban Slum Environment. PLoS Neglected Tropical Diseases. 2014;8(12).

[36] Panti-May JA, Carvalho-Pereira TSA, Serrano S, et al. A Two-Year Ecological Study of Norway Rats (Rattus norvegicus) in a Brazilian Urban Slum. PLoS ONE. 2016;11(3).

[37] Costa F, Wunder EA, de Oliveira D, et al. Patterns in Leptospira shedding in Norway rats (Rattus norvegicus) from Brazilian slum communities at high risk of disease transmission. PLoS Neglected Tropical Diseases. 2015;9(6).
[38] Porter FH, Costa F, Rodrigues G, et al. Morphometric and demographic differences between tropical and temperate Norway rats (Rattus norvegicus). Journal of Mammalogy. 2015;96(2):317-323.
[39] Peig J, Green AJ. New perspectives for estimating body condition from mass/length data: the scaled mass index as an alternative method. Oikos. 2009;118(12):1883-1891.
[40] McCallum ES, Cerveny D, Fick J, et al. Slow-Release Implants for Manipulating Contaminant Exposures in Aquatic Wildlife: A New Tool for Field Ecotoxicology. 2019.
[41] Cerveny D, Grabic R, Grabicová K, et al. Neuroactive drugs and other pharmaceuticals found in blood plasma of wild European fish. Environment International. 2021;146.
[42] Grabic R, Fick J, Lindberg RH, et al. Multi-residue method for trace level determination of pharmaceuticals in environmental samples using liquid chromatography coupled to triple quadrupole mass spectrometry. Talanta. 2012;100:183-195.
[43] Lindberg RH, Östman M, Olofsson U, et al. Occurrence and behaviour of 105 active pharmaceutical ingredients in sewage waters of a municipal sewer collection system. Water Research. 2014;58:221-229.
[44] Athanazio DA, Silva EF, Santos CS, et al. Rattus norvegicus as a model for persistent renal colonization by pathogenic Leptospira interrogans. Acta Tropica. 2008;105(2):176-180.
[45] Chagas-Junior AD, McBride AJA, Athanazio DA, et al. An imprint method for detecting leptospires in the hamster model of vaccine-mediated immunity for leptospirosis. Journal of Medical Microbiology. 2009;58(12):1632-1637.
[46] Chagas-Junior AD, da Silva CLR, Soares LM, et al. Detection and quantification of leptospira interrogans in hamster and rat kidney samples: Immunofluorescent imprints versus real-time PCR. PLoS ONE. 2012;7(2).
[47] Bustin SA, Benes V, Garson JA, et al. The MIQE guidelines: minimum information for publication of quantitative real-time PCR experiments. Clin Chem. 2009;55(4):611-22.
[48] Team RC. R: A language and environment for statistical computing. R Foundation for Statistical Computing; 2023.
[49] Egorov AI, Converse R, Griffin SM, et al. Environmental risk factors for Toxoplasma gondii infections and the impact of latent infections on allostatic load in residents of Central North Carolina. BMC Infectious Diseases. 2018;18(1).
[50] Heyman P, Baert K, Plyusnina A, et al. Serological and genetic evidence for the presence of Seoul hantavirus in Rattus norvegicus in Flanders, Belgium. Scandinavian Journal of Infectious Diseases. 2009;41(1):51-56.
[51] Kümmerer K. Pharmaceuticals in the environment. Annual Review of Environment and Resources. 2010;35:57-75.
[52] Rasheed T, Bilal M, Nabeel F, et al. Environmentally-related contaminants of high concern: Potential sources and analytical modalities for detection, quantification, and treatment. Environment International: Elsevier Ltd; 2019. p. 52-66.
